# How to perform the dusting technique for calcium oxalate stone phantoms during Ho:YAG laser lithotripsy

**DOI:** 10.1186/s12894-018-0417-5

**Published:** 2018-11-13

**Authors:** Jeong Woo Lee, Min Gu Park, Sung Yong Cho

**Affiliations:** 1 Department of Urology, Dongguk University Ilsan Hospital, Dongguk University College of Medicine, 27, Dongguk-ro, Ilsandong-gu, Goyang-si, Gyeonggi-do 410-773 Republic of Korea; 2 Department of Urology, Seoul Paik Hospital, Inje University College of Medicine, 9, Mareunnae-ro, Jung-gu, Seoul 100-032 Republic of Korea; 3 Department of Urology, Seoul Metropolitan Government-Seoul National University Boramae Medical Center, Seoul National University College of Medicine, 20, Boramae-ro 5-Gil, Dongjak-gu, Seoul 156-707 Republic of Korea

**Keywords:** Calcium oxalate, Dusting, Energy, Ho:YAG laser, Lithotripsy

## Abstract

**Background:**

To determine the most efficacious setting of Holmium:yttrium-aluminum-garnet (Ho:YAG) laser with a maximum power output of 120 W with in vitro phantom-stone dusting technique.

**Methods:**

A laser was used to treat two 4 × 3 × 3 mm^3^ sized phantom stones in 5 mL syringes with 1 mm-sized holes at the bottom. According to the pulse width (short 500, middle 750, long pulse 1000 μsec), maximal pulse repetition rates from 50 to 80 Hz were tested with pulse energy of 0.2, 0.4, 0.5, and 0.8 J. Six times of the mean dusting times were measured at each setting. Dusting was performed at continuous firing of the laser until the stones become dusts < 1 mm.

**Results:**

The mean Hounsfield unit of phantom stones was 1309.0 ± 60.8. The laser with long pulse generally showed shorter dusting times than short or middle pulse width. With increasing the pulse energy to 0.5 J, the dusting time decreased. However, the pulse energy of 0.8 J showed longer dusting times than those of 0.5 J. On the post-hoc analysis, the pulse energy of 0.5 J, long pulse width, and the repetition rates of 70 Hz demonstrated significantly shorter dusting times than other settings.

**Conclusions:**

The results suggest that long pulse width with 0.5 J and 70 Hz would be the most efficacious setting for dusting techniques of plaster stone phantoms simulating calcium oxalate stones using the 120 W Ho:YAG laser.

## Background

Laser lithotripsy has remained the first-line treatment option for urinary stones with technical advancements in dedicated endoscopes, instruments, and accessories [1–3]. Recent investigations demonstrated high success rates and low complication rates of the minimally invasive surgical techniques using the Holmium:yttrium-aluminum-garnet (Ho:YAG) laser, especially in miniaturized percutaneous nephrolithotomy and retrograde intrarenal surgery [4–7]. The pulsed Ho:YAG laser has become one of the main lithotripters along with the ultrasonic or pneumatic lithotripter [2].

Laser efficacy during lithotripsy is essential to obtain the maximal surgical efficacy and excellent surgical outcome. The efficacy of Ho:YAG laser-mediated stone fragmentation is better with increased energy per pulse and reduced pulse width, but not consistently with pulse repetition rates with a power output of 10~ 20 W [8–10]. Meanwhile, stone dusting with low pulse energy and high pulse repetition rates reduces the size of fragmented stones until they become dusts, which improves stone clearance [8]. This is because the Ho:YAG laser produces less retropulsion from the fiber tip in the lower power energy, which affects the surgical efficacy.

The recent development of the high-power output 120 W Ho:YAG laser system has provided surgeons with additional options for stone dusting, courtesy of increased pulse repetition rates from 50 to 80 Hz and three different options of pulse width from 500 to 1000 μsec. However, there is no consensus of the optimal laser setting for stone dusting. To provide clarity, we investigated the impact of pulse energy, width, and repetition rates on the dusting efficacy of phantom stones in vitro using the 120 W Ho:YAG laser system. The aim was to determine the most efficacious laser setting for stone dusting.

## Methods

The authors sought to determine the influence on the dusting efficacy according to each setting value of the hand-held optical fiber of Ho:YAG laser pulse energy (pulse width) and the repetition rate based on each pulse width.

### Laser system and parameters

The experiments were performed using a 2.1 μm emitting Lumenis VersaPulse PowerSuite Holmium (Ho:YAG) surgical laser 120H® (Lumenis Ltd., Israel) with a maximum power output of 120 W for fibers with core diameters of 200 μm. Pulse widths were short (500 μsec), middle (750 μsec), and long (1000 μsec). The maximal pulse repetition rates were 50, 70, and 80 Hz. The pulse energies were 0.2, 0.4, 0.5, and 0.8 J. The maximal repetition rates differed according to the pulse width and pulse energy.

### Stone sample preparation

**Fig. 1:**
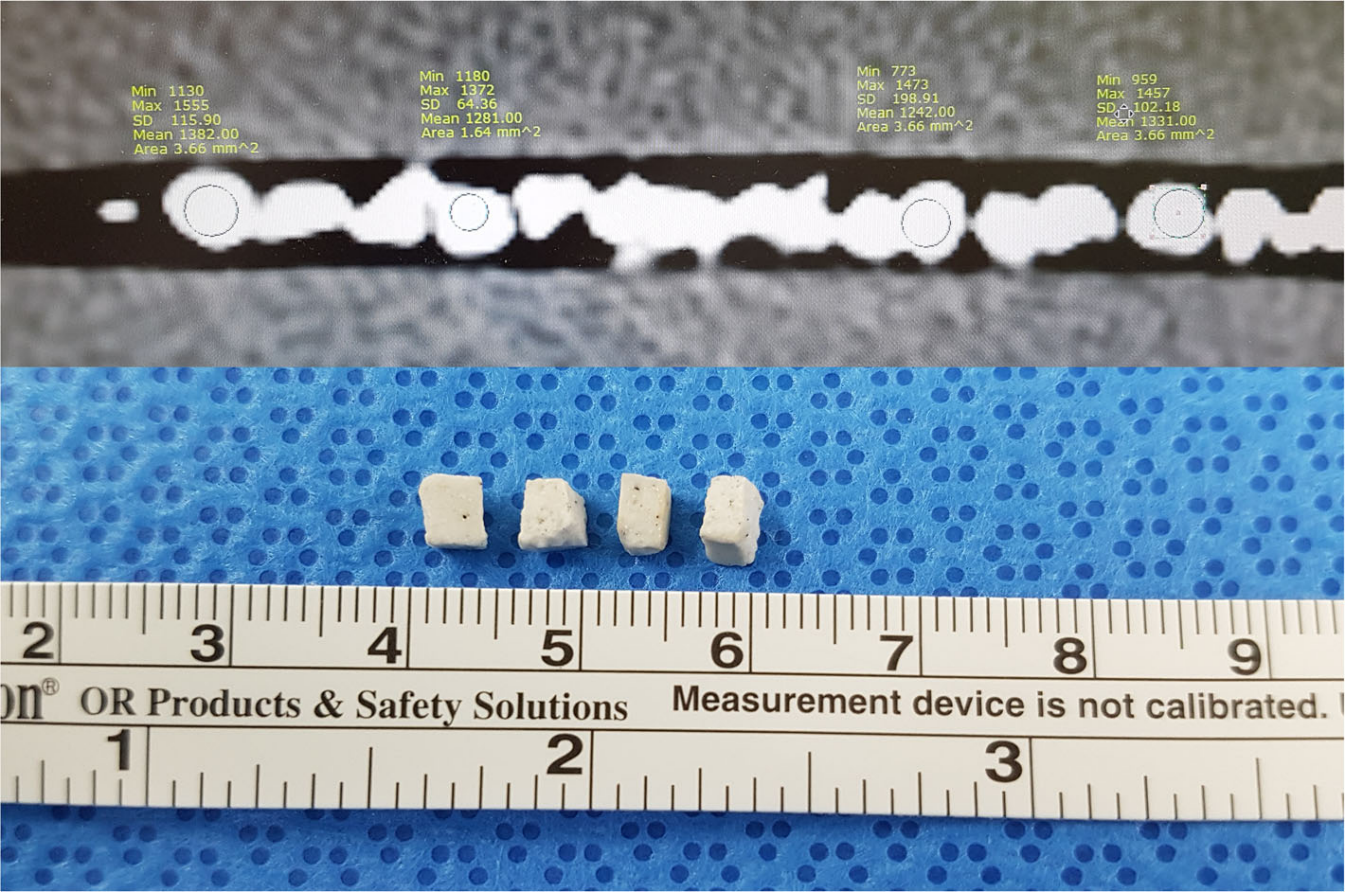
**a** Stone density measured in the computed tomography scan images. **b** Each cubical stone of 4x3x3 mm^3^

The molded plaster phantom stones were obtained from SINI Inc. (Ui-Wang, Gyeonggi-do, Korea) (Fig. 1). The stone density mimics the hardness of human calcium oxalate monohydrate calculi, consistent with a prior study [4]. Two calculi were used for each laser experiment. The stone size was cut up into equal cubical pieces of 4 × 3 × 3 mm^3^.

### Hand-held dusting techniques

**Fig. 2:**
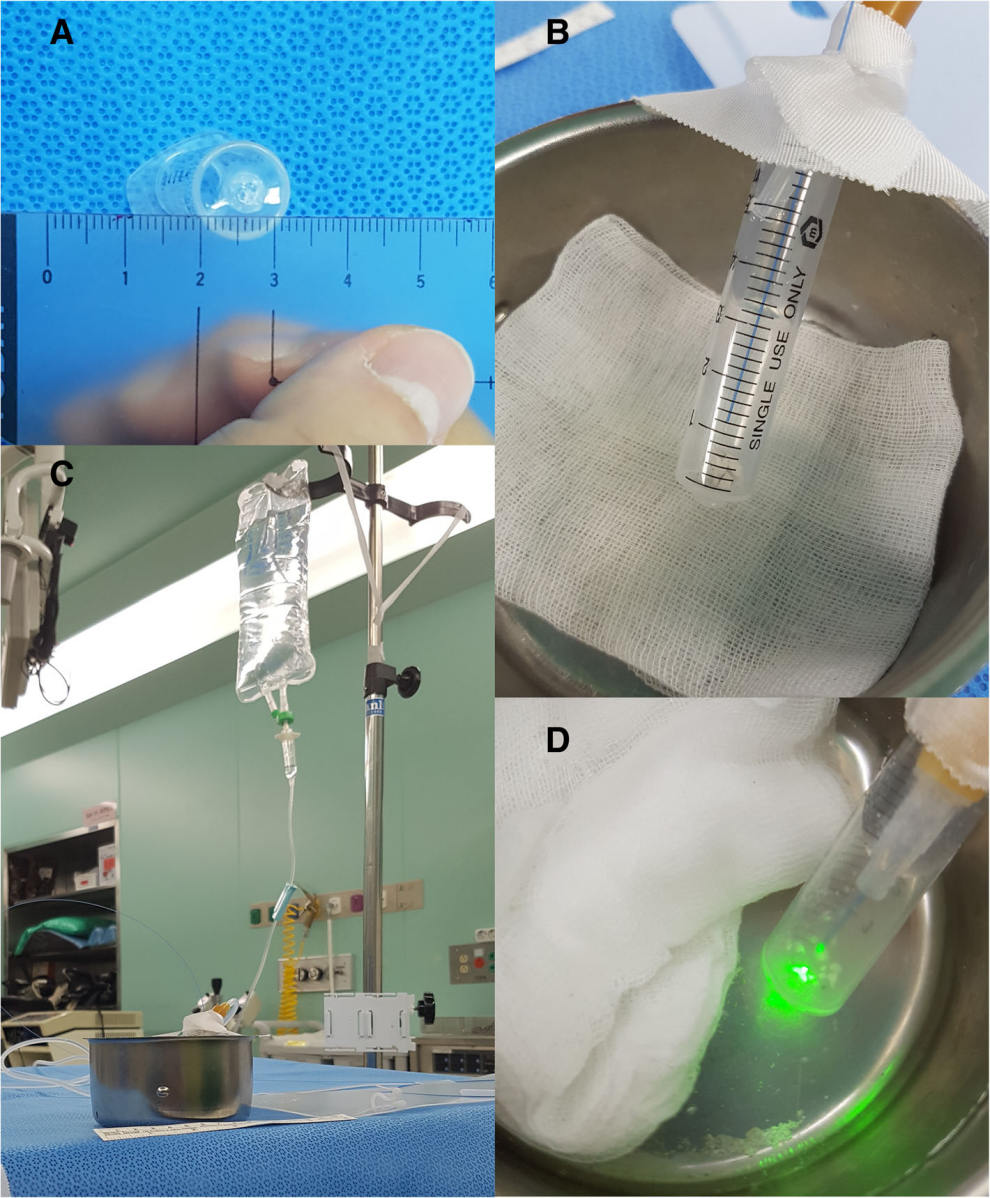
**a** A 1 mm-sized hole at the bottom of the syringe for fragmented particles to go out. **b** A laser fiber was positioned 1–2 mm away from the phantom stones when the dusting technique starts. **c** Irrigation fluid at the height of 40cmH_2_O to mimic the real practice situation. **d** Dusts < 1 mm went out of the syringe during laser firing. When the all particles disappear in the syringe, the duration of dusting was checked by a stop-watch

Only freshly cleaved 200 μm fibers were used. The fiber tip was positioned 1 to 2 mm from the phantom stone by the investigator (Cho SY). The 5 ml syringes had a 1 mm-sized hole at the bottom where stone dust exited the syringe into a pan (Fig. 2). The irrigation pressure was set to 40 cmH_2_O from the phantom stones. Dusting was performed with continuous firing of the laser until the stones became a dust with a particle size < 1 mm. The dusting time was defined from the initiation of laser firing to the formation of this dust.

### Statistical analyses

All parameters represented the mean value ± standard deviation (percentage). Comparative results were analyzed using independent t-test or Mann-Whitney U test between the two groups and Kruskal-Wallis test among the groups. Post-hoc analysis with Tukey’s honestly significant difference test was performed. Categorical variables were analyzed by Chi-square and Fisher’s exact test. Statistical significance was considered at *P* < .05. Statistical analyses were performed by the statistical software SPSS version 20 (IBM, Armonk, NY) and R version 3.0.1 (http://www.r-project.org).

## Results

The mean Hounsfield unit was 1309.0 ± 60.8. The mean dusting time was determined from six measurements of each study criterion given. The results are summarized in Table 1. The highest repetition rate was 70 Hz with long and middle pulse widths and pulse energies of 0.2, 0.4, and 0.5 J, and 80 Hz with short pulse width and pulse energies from 0.2 to 0.5 J. The highest repetition rate was 50 Hz for 0.8 J of pulse energy for each pulse width.

**Table 1:** Dusting time (sec) according to each laser setting

Dusting time (sec)	Hz	Test	0.2 J	Hz		0.4 J	Hz		0.5 J	Hz		0.8 J
Short pulse	80	1	1120	80	1	720	80	1	540	50	1	600
		2	1080		2	800		2	660		2	780
		3	1560		3	750		3	900		3	720
		4	1440		4	960		4	540		4	910
		5	1350		5	1000		5	600		5	800
		6	1470		6	750		6	580		6	760
	Mean ± S.D		1336.7 ± 195.6	Mean ± S.D		830.0 ± 119.7	Mean ± S.D		636.7 ± 136.5	Mean ± S.D		761.7 ± 101.7
Middle pulse	70	1	1140	70	1	780	70	1	360	50	1	780
		2	1250		2	900		2	480		2	700
		3	1080		3	820		3	400		3	650
		4	1360		4	990		4	500		4	660
		5	1240		5	800		5	420		5	590
		6	1180		6	700		6	410		6	660
	Mean ± S.D		1208.3 ± 97.7	Mean ± S.D		831.7 ± 100.9	Mean ± S.D		428.3 ± 52.3	Mean ± S.D		673.3 ± 63.1
Long pulse	70	1	1140	70	1	540	70	1	300	50	1	540
		2	1260		2	600		2	350		2	500
		3	1050		3	480		3	280		3	620
		4	1300		4	580		4	320		4	600
		5	1220		5	600		5	350		5	500
		6	1200		6	900		6	300		6	480
	Mean ± S.D		1195.0 ± 89.4	Mean ± S.D		616.7 ± 146.1	Mean ± S.D		316.7 ± 28.8	Mean ± S.D		540.0 ± 58.0

The long pulse width generally produced shorter dusting times than short or middle pulse widths. As the pulse energy increased to 0.5 J, the dusting time decreased. However, the pulse energy of 0.8 J produced a longer dusting time than pulse energy of 0.5 J.

**Fig. 3:**
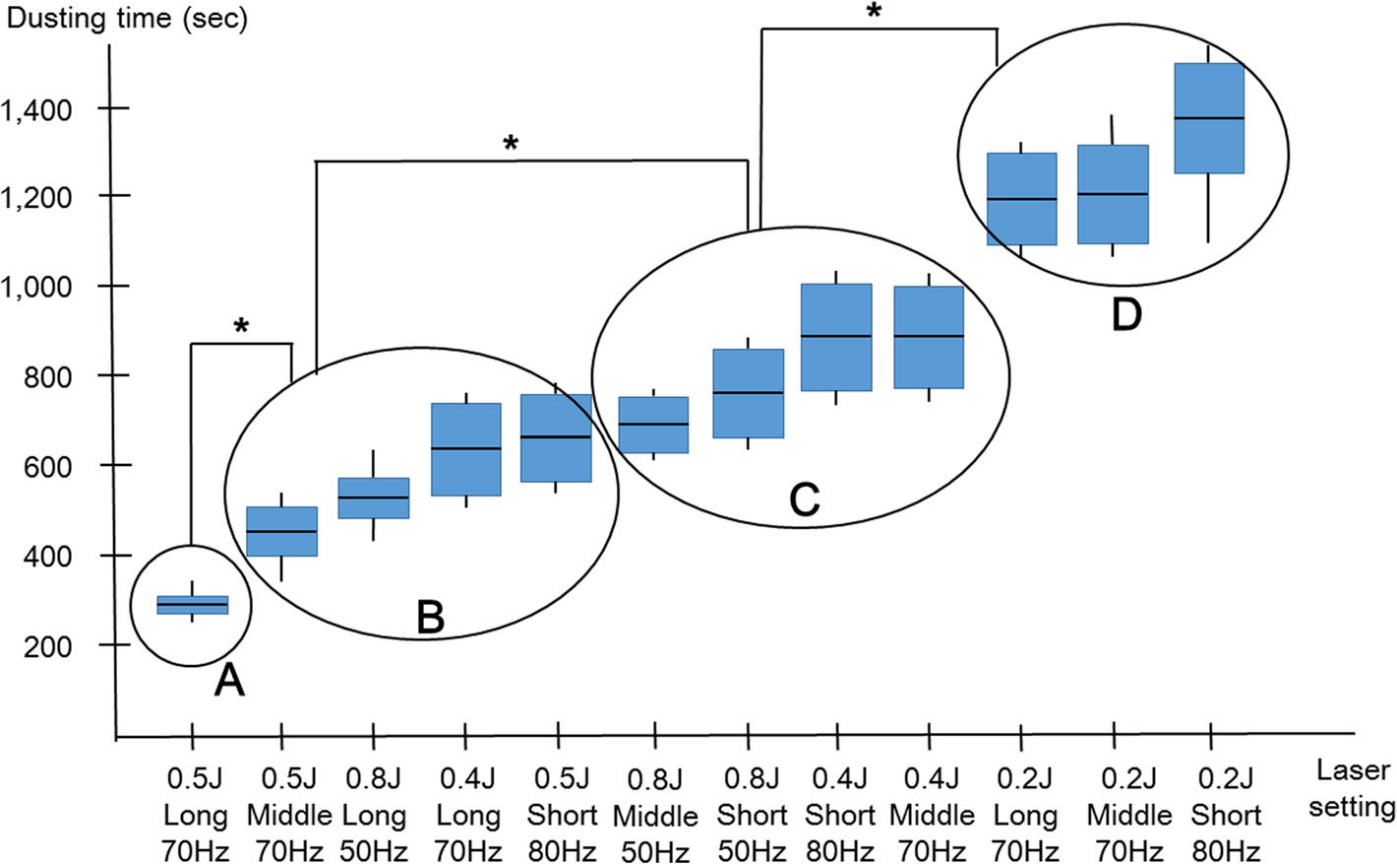
Post-hoc analysis to compare the mean dusting time per each setting and across the groups **a** (0.5 J, a long pulse width, and 70 Hz), **b** (0.5 J (middle and short pulse widths), **c** (0.4 and 0.8 J, middle or short pulse width), and **d** (0.2 J groups)

Figure 3 depicts results of a post-hoc analysis of the mean dusting time measured at each setting. Pulse energy of 0.5 J, a long pulse width, and a repetition rate of 70 Hz proved to be the most efficacious dusting setting (Group A). Group B included pulse energy of 0.5 J (middle and short pulse widths) and 0.4 J or 0.8 J (long pulse width). Group C included pulse energies of 0.4 J and 0.8 J with middle or short pulse width. Group D comprised pulse energy of 0.2 J regardless of pulse width and repetition rate.

## Discussion

The pulsed Ho:YAG laser is used predominantly with flexible ureterorenoscopic and miniaturized percutaneous devices. This laser has become the preferred lithotripter in clinical use over the past two decades [2]. The maximal efficacy of laser lithotripsy techniques, mainly stone fragmentation and dusting, are essential to improve surgical outcomes. The efficacy of lithotripsy obtained using the Ho:YAG laser depends on laser settings that include energy per pulse, pulse width, and pulse repetition rates [8]. Factors that favor the fragmentation efficacy of the Ho:YAG laser with a power output of 10~ 20 W are increased pulse energy and reduced pulse width [8–10]. Stone dusting is a recently established outcome of Ho:YAG laser use. Dusting is routinely performed with a low pulse energy and high pulse repetition rate to obtain maximum stone clearance. A Ho:YAG laser system with a maximum power output of 120 W was recently developed, which enables the surgeon to choose increased pulse repetition rates of 50 Hz or 80 Hz according to the pulse energy. Additionally, this new device has three different options of pulse width (short, middle, and long pulse of 500, 750, and 1000 μsec, respectively). Few investigations have assessed the optimal settings of this laser system. The present study involving in vitro reproducible experiments with phantom stones was done to define the most efficient laser setting for stone dusting.

The ideal for stone dusting during Ho:YAG lithotripsy is to use a setting that produces maximal fragmentation efficacy. The aim is to transform stone fragments into dust particles < 1 mm in size. Previous investigations explored the effect of various pulse energy of the Ho:YAG laser for stone fragmentation [11–13]. Increased pulse energy increases fragmentation power but increases retropulsion for the fragmented stones. Increased retropulsion may induce less energy transmission to stones and lower repetition rates, which may result in less fragmentation efficacy [14]. Low pulse energy (0.2 J) produces small fragment debris and less retropulsion at a slower fragmentation rate [11]. Presently, a pulse energy of 0.5 J and 70 Hz repetition rate with a long pulse was the most appropriate setting for stone dusting of plaster stones representing calcium oxalate monohydrate stones. This may be because retropulsion is significant in determining stone dusting efficacy. A low pulse energy of 0.2 or 0.4 J may be not efficacious to fragment phantom stones with a mean Hounsfield unit of 1309.0.

The association between pulse width and stone fragmentation efficacy has been studied in vitro [8, 9, 14–17]. In one study, short pulse width (120–190 μsec) produced equivalent fragmentation effectiveness, but more retropulsion compared to long pulse width (210–350 μsec) [15]. A ureter and caliceal model was used to demonstrate that a pulse width of 700 μsec provided less retropulsion and more effective stone fragmentation compared to a pulse width of 350 μsec [14]. In contrast, in an in vitro impacted and immobile phantom stones model, reduction of the pulse width from 700 to 350 μsec increased the fragmentation effectiveness of a Ho:YAG system with 10 W power [9]. In the present study, the mean dusting time decreased with increasing pulse width from 500 to 1000 μsec. The long pulse width (1000 μsec) provided the most effective stone dusting at a pulse energy ≥0.4 J.

Pulse repetition rates may not be critical to fragmentation efficacy [10, 11]. In these studies, the mean dusting time did not differ significantly at a pulse repetition rate of 70 and 80 Hz. These findings support the view that energy per pulse and pulse width, rather than pulse repetition rate, are more closely associated with stone fragmentation and stone dusting.

The present results might support the following ‘ideal’ settings of the Ho:YAG laser in stone dusting. The energy should be as low as possible to minimize retropulsion, while being powerful enough to break down the targeted stones. A longer pulse width is better than a shorter width. Higher repetition rates may be better than the lower ones.

Evidence about the dusting efficacy during stone surgery with the 120 W Ho:YAG laser system is limited. During laser lithotripsy, dusting technique usually needs the laser setting of low-pulse energy and high frequency [18]. A recent investigation assessed surgical outcomes of dusting technique in 82 renal units of 71 patients utilizing 120 W Ho:YAG laser with 200-μm fibers [19]. The mean stone size was 12.5 ± 8.7 mm and the mean Hounsfield unit was 993 ± 353. The laser setting for hard stones (> 1000 HU) during dusting technique was pulse energy of 0.3 J, 70 Hz repetition rates and short pulse width mode. For soft stones (< 1000 HU), the laser setting was pulse energy of 0.2 J, 80 Hz repetition rates and short pulse width mode. Although there were no direct comparative results between short and long pulse width modes, the complete stone free rate was 39% and < 2 mm residual fragments were identified in 69%. Another important point is the heat generation during laser lithotripsy. There have been few studies on thermal effects in terms of injury to adjacent organs during dusting technique with the 120 W Ho:YAG laser system. The authors did not measure fluid temperature during continuous firing of the laser. However, continuous irrigation with cool normal sline prevented overgeneration of heat during experiments. Further laboratory studies or clinical trials are needed to confirm the most efficacious and safe setting for dusting technique with the 120 W Ho:YAG laser system.

This study has some limitations. The experiments were not performed to mimic minor calyces of the human kidneys. So, the results do not reflect the situation in which a fragmented stone might migrate from one to another calyx. Phantom stones were previously reported to provide an adequate model to evaluate efficacy of stone fragmentation and retropulsion of Ho:YAG laser setting [8–17]. The authors used a single kind of phantom stone, which mimicked human calcium oxalate monohydrate calculi. The optimal stone fragmentation can be achieved according to helical/snail schema and the present study could not show the effect of stone retropulsion. In addition, only straightened laser fibers of 200 μm were used. Further studies are needed to determine the appropriate laser settings for other clinically possible situations including different kinds of stones.

## Conclusions

In vitro reproducible experiments with phantom stones mimicking calcium oxalate monohydrate calculi demonstrates that a pulse energy of 0.5 J, long pulse width, and a repetition rate of 70 Hz provides the most efficacious dusting with the high-power output 120 W Ho:YAG laser in combination with a 200-μm fiber. The findings do not apply to other types of human calculi, but still have value in clinical practice.

## Abbreviation

Ho:YAG: Holmium:yttrium-aluminum-garnet
